# Impact of residual moderate mitral regurgitation after transcatheter edge-to-edge repair on long-term survival: insights from a multicenter cohort study

**DOI:** 10.3389/fcvm.2026.1720322

**Published:** 2026-02-18

**Authors:** Felix Ausbuettel, Georgios Chatzis, Harald Schuett, Sebastian Barth, Sebastian Kerber, Bernhard Schieffer, Christian Waechter, Ulrich Luesebrink

**Affiliations:** 1 Department of Cardiology, University Hospital Marburg, Baldingerstrasse Marburg, Marburg, Germany; 2 Department of Medicine, Philipps-University Marburg, Marburg, Germany; 3 Department of Cardiology, Cardiovascular Center Bad Neustadt/Saale, Bad Neustadt an der Saale, Germany

**Keywords:** MitraClip, mitral regurgitation, mTEER, PASCAL, residual severity, transcatheter edge-to-edge mitral valve repair

## Abstract

**Background:**

Transcatheter edge-to-edge mitral valve repair (mTEER) is an established treatment for patients with mitral regurgitation (MR) at prohibitive surgical risk. However, predictors of optimal MR reduction remain insufficiently defined.

**Methods:**

We retrospectively analyzed all patients who underwent mTEER treatment at four German cardiac centers between 2011 and 2022. Patients were categorized by residual MR ≤ I° or II°. Univariable and multivariable logistic regression analyses revealed predictors of residual MR ≤ I°. Long-term survival was assessed via Kaplan–Meier analysis and adjusted through propensity-score matching (PSM).

**Results:**

Among 821 patients, 724 (88.2%) achieved residual MR ≤ I°, whereas 97 (11.8%) achieved residual MR of II°. Patients with residual MR of II° were younger, exhibited higher morbidity, and required longer procedures with more clips. Age <65 years, prior implantable cardioverter defibrillator (ICD), and left ventricular end-diastolic diameter >62 mm served as inverse predictors of residual MR ≤ I° in univariable analysis. In contrast, only age <65 years remained a significant inverse predictor of residual MR ≤ I° in the multivariable model (odds ratio 0.39, 95% confidence interval 0.19–0.84, *p* = 0.01). Long-term survival did not differ significantly in the overall cohort between patients with residual MR ≤ I° and those with residual MR of II° before or after PSM.

**Conclusion:**

Residual MR of II° occurred in 12% of patients and was more common in younger, comorbid individuals with complex MR anatomy. While survival was unaffected in the overall cohort, age <65 years emerged as a key predictor of suboptimal MR reduction. These findings highlight the need for critical patient selection and may inform individualized treatment strategies in contemporary mTEER practice.

## Graphical Abstract

**Figure:**
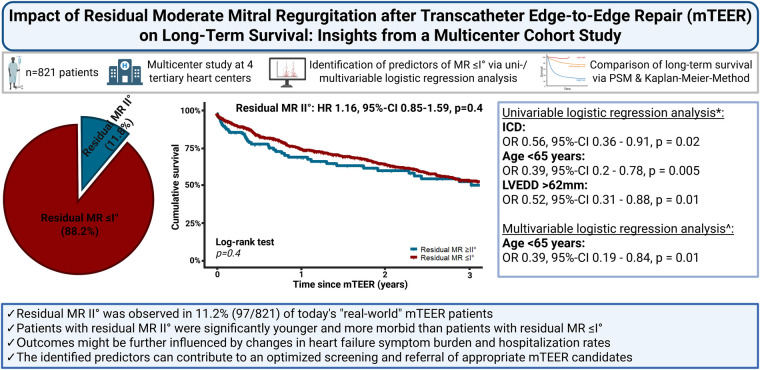
*Univariable logistic regression analysis of independent predictors of residual MR ≤ I° after mTEER. ^Multivariable logistic regression analysis of the identified independent predictors of residual MR ≤ I° in the univariable logistisc regression analysis. HR, hazard ratio; ICD, implantable cardioverter defibrillator; LVEDD, left ventricular enddiastolic diameter; MR, mitral valve regurgitation; mTEER, transcatheter edge-to-edge mitral valve repair; OR, odds ratio; PSM, propensity score matching; 95%-CI, 95% confidence interval.

## Introduction

The continously increasing prevalence of congestive heart failure and the ongoing demographic changes in the general population of Western industrialized nations (1, 2) have led to severe mitral regurgitation (MR) becoming one of the most common types of valvular heart disease (3, 4). Although it was initially applied only to a limited number of high-risk patients (5), the transcatheter edge-to-edge mitral valve repair (mTEER) procedure has gained considerable importance in the therapeutic management of symptomatic patients deemed unsuitable for surgical mitral valve repair (4). This shift is primarily attributed to its favorable safety and effectiveness profile (6–9). Consequently, a continuous increase in the number of mTEER procedures is to be expected in the future (5).

Despite recent therapeutic advancements in the contemporary mTEER era, residual moderate-to-severe MR is still observed in 4.7%–30% of patients at the postprocedure stage (10–14). While the underlying factors contributing to insufficient reduction in MR severity can be multifaceted, recent evidence has demonstrated a negative impact of residual moderate-to-severe MR on long-term survival after mTEER (10, 11, 13–16).

On the basis of the available data, it seems reasonable to aim for the greatest possible reduction in MR to mild or minimal residual severity (≤I°) during mTEER and, in particular, to refer preferably those patients for mTEER in whom this therapeutic goal appears achievable. However, the interpretation of the available studies is significantly restricted by inconsistent definitions of residual MR severity cut-off values (10–13, 15, 17). We therefore aimed to identify preprocedural predictors of successful reduction in MR to mild or minimal severity. Moreover, we intended to elucidate the so far unclarified impact of residual moderate MR (II°) on long-term survival, as the negative effect on survival after mTEER has primarily been demonstrated for moderate-to-severe residual MR (10, 11, 15, 16).

## Materials and methods

In this multicenter cohort study, all patients who underwent mTEER treatment at four German tertiary heart centers between October 2011 and December 2022 were included for further analysis. Patients were evaluated for mTEER if they remained symptomatic despite optimal medical therapy for congestive heart failure, if anatomical suitability for mTEER was confirmed by echocardiography, and if they were deemed unsuitable candidates for surgical mitral valve repair. The echocardiographic criteria for the anatomical and morphological suitability of MR were conformed with those of the Cardiovascular Outcomes Assessment of the MitraClip Percutaneous Therapy for Heart Failure Patients with Functional Mitral Regurgitation (COAPT) study for functional MR (6) and those of the Endovascular Valve Edge-to-Edge Repair (EVEREST) II study for degenerative MR (7). Surgical unsuitability was determined by an interdisciplinary heart team, consisting of cardiac surgeons, and interventional and conservative cardiologists. The mTEER procedure was performed using either the PASCAL™ system (Edwards Lifesciences, Irvine, CA, USA) or the MitraClip© system (Abbott Vascular, Santa Clara, CA, USA) under either analgosedation or general anesthesia, as previously described (18–20). Procedural success was defined as follows: a reduction in MR severity to a residual MR of II° or MR of ≤I° in the absence of mitral valve stenosis with a mean gradient >5 mmHg across the valve. Patients whose mTEER procedure had to be aborted prematurely because of insufficient grasping of the leaflets were excluded from the analysis. Major adverse cardiovascular and cerebrovascular events (MACCEs) were reported in accordance with the recommendations of the Mitral Valve Academic Research Consortium (MVARC) (21).

The echocardiographic assessment of MR was conducted prior to mTEER by the designated laboratories, thereby employing semiquantitative and quantitative parameters as outlined in the prevailing guidelines (4). The graduation of residual MR was performed at the time of early post-interventional echocardiography prior to hospital discharge. The criteria applied for the graduation of residual MR were listed in Supplementary Table S1 according to previous publications (22–24). Patients who were included in the analysis were allocated to the respective cohorts with residual MR severity ≤I° or residual MR severity II° after mTEER. The primary endpoint of the analysis was all-cause mortality during the follow-up period after mTEER.

### Statistical analysis

Continuous normally distributed variables are presented as the means with standard deviations (SDs). Continuous nonnormally distributed variables were presented as the medians with interquartile ranges (IQRs, 25th–27th percentiles). The presence of a normal distribution was confirmed via the Shapiro–Wilk test. Categorical variables were presented as absolute and relative frequencies (%). Differences between cohorts were assessed for statistical significance on the basis of Student's *t*-test for normally distributed continuous variables and the Wilcoxon rank sum test for nonnormally distributed continuous variables. For categorical variables, the chi-squared test was employed if the expected cell size was ≥20, and Fisher's exact test was applied if the expected cell size was <20. A two-sided *p*-value ≤ 0.05 was considered to indicate statistical significance.

Propensity score matching (PSM) was implemented to adjust clinical parameters with significant differences between the cohorts and parameters with a confirmed influence on mortality, thereby enabling an adjusted mortality analysis. For this purpose, a PSM was performed using the nearest neighbor method. The propensity scores were estimated using a generalized linear model using logistic regression. The caliper, defined as the maximum difference between the propensity scores of the matched patients, was set to a value of 0.2. The PSM ratio was selected on the basis of the cohort of patients with residual MR of II° that revealed the lowest number of patients. In the present cohort, a 1:6-matching ratio was applied as the maximum possible ratio for adjusting the variables. The matching parameters were selected on the basis of clinical variables with significant differences between the cohorts and mortality predictors identified in the present cohort using univariable and multivariable Cox regression analyses. The chosen matching parameters were age, heart failure symptoms of New-York-Heart-Association (NYHA) class IV, previous implantation of an implantable cardioverter defibrillator (ICD), and the respective etiology of MR. Long-term mortality between patients with and without residual MR ≤ I° was analyzed using the Kaplan–Meier method, and differences were compared via the log-rank test. The quality of covariate balancing was assessed by calculating standardized mean differences (SMDs), with an SMD ≤ 0.2 defined as sufficient balancing and an SMD of 0.21–0.5 defined as moderate balancing (25).

To identify predictors of residual MR ≤ I° after mTEER, a univariable logistic regression analysis was performed. All variables with a *p*-value < 0.1 were then incorporated into the multivariable logistic regression model. For continuous variables, a threshold value was also presented for which a significant correlation with the dependent variable could be identified.

All statistical analyses were performed using R Studio V4.4.2 (R Foundation for Statistical Computing, Vienna, Austria) and the “rcmdr”, “survival”, “survminer”, “dplyr”, “tableone”, “My.stepwise”, “stddiff” and “MatchIt” packages. The presented graphics and the graphical abstract were designed using BioRender.com (Science Suite Inc., Toronto, Ontario, Canada).

### Missing data

In the case of an insufficient follow-up of <30 days, a mortality query was performed via the respective registration office. Despite the increased efforts, 6.7% (55/821) of patients were lost to follow-up during the observation period. The subsequent analysis revealed no evidence of an informative absence or of a significant impact of patients who were lost to follow-up on the results. An overview of the clinical characteristics of patients with and without loss to follow-up is provided in Supplementary Table S2.

## Results

**Figure 1:**
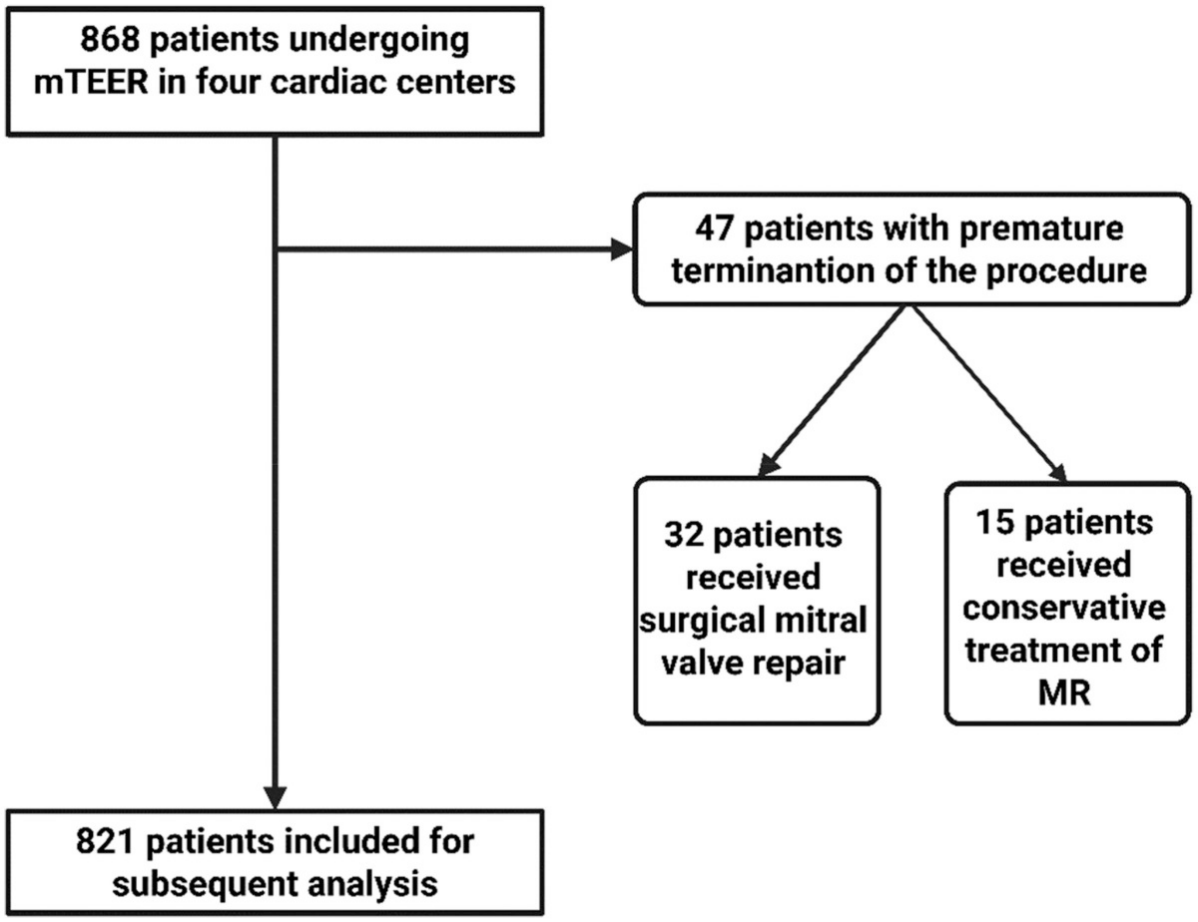
Flowchart for the inclusion and exclusion of patients undergoing mTEER. MR, mitral valve regurgitation; mTEER, transcatheter edge-to-edge mitral valve repair.

A total of 868 patients underwent mTEER treatment in the four German heart centers during the observational period. The procedure had to be terminated prematurely in 47 patients (5.4%), 32 of whom (3.7%) subsequently underwent surgical mitral valve repair. The remaining 15 patients (1.7%) were treated conservatively. The remaining 821 patients were enrolled for further analysis. A flowchart showing the inclusion and exclusion of patients is shown in Figure 1.

### Clinical cohort characteristics and short-term outcomes

Within the investigated cohort, most patients 88.2% experienced a reduction in MR to ≤I°, and the remaining 11.8% (97/821) exhibited residual MR of II° after mTEER. Patients exhibiting residual MR ≤ I° were significantly older and exhibited significantly lower NYHA classes than did patients with residual MR of II°. Furthermore, a significantly lower rate of ICD implantation was observed among patients with residual MR ≤ I°. With respect to procedural characteristics, patients with residual MR ≤ I° had significantly shorter procedure times and lower clip implantation rates than patients with residual MR of II°. Despite the absence of statistical significance in the comparison of periprocedural complications and MACCE across the cohorts, a notable finding emerged from the analysis: Specifically, patients exhibiting residual MR ≤ I° demonstrated a significantly reduced postinterventional hospitalization duration. The clinical and procedural characteristics of the cohorts are delineated in Table 1, and the respective data concerning the short-term outcomes are provided in Table 2.

**Table 1:** Clinical and procedural characteristics of patients with or without residual MR ≤ I° following mTEER before and after propensity-score-matching.

Variable	Overall cohort	Before Propensity-Score-Matching				After Propensity-Score-Matching			
		Residual MR II° after mTEER	Residual MR ≤ I° after mTEER	*p*-value^#^	SMD^◊^	Residual MR II° after mTEER	Residual MR ≤ I° after mTEER	*p*-value^#^	SMD^◊^
	(*n* = 821)	(*n* = 97	(*n* = 724)			(*n* = 97)	(*n* = 582)		
Age (years)	78 ± 8	77 ± 10	79 ± 8	**0** **.** **03**	0.21	77 ± 10	77 ± 8	0.4	0.1
Male sex	62.6% (514)	61.9% (60)	62.7% (454)	1	0.018	61.9% (60)	65.3% (380)	0.6	0.07
euroSCORE II (%)*	16 (8.4–28.3)	18.6 (10–35.2)	15 (8–28)	0.08	0.15	18.6 (10–35.2)	16 (8–29)	0.2	0.07
STS Risk Score (%)*	6.6 (4–12)	7.3 (4.2–15.2)	6.6 (3.9–11.6)	0.2	0.13	7.3 (4.2–15.2)	6.7 (4–12.4)	0.2	0.1
BMI (kg/m^2^)	27 ± 5	27.1 ± 5.4	27 ± 5	0.7	0.04	27.1 ± 5.4	27 ± 5	0.8	0.04
NYHA class I	0.1% (1)	0% (0)	0.1% (1)	**0** **.** **045**	0.27	0% (0)	0.2% (1)	0.07	0.2
NYHA class II	3.2% (26)	6.2% (6)	2.7% (20)			6.2% (6)	2.4% (14)		
NYHA class III	75.6% (621)	67% (65)	76.8% (556)			67% (65)	73.7% (429)		
NYHA class IV	21.1% (173)	25.8% (25)	20.4% (148)			25.8% (25)	23.7% (138)		
COPD	17.8% (146)	14.4% (14)	18.2% (132)	0.4	0.1	14.4% (14)	19.8% (115)	0.3	0.14
CAD	62.2% (511)	60.8% (59)	62.4% (452)	0.8	0.03	60.8% (59)	64.4% (375)	0.5	0.08
Prior CAB-OP	27.8% (228)	33% (32)	27.1% (196)	0.3	0.13	33% (32)	28.7% (167)	0.4	0.09
Prior PCI	54% (443)	49.5% (48)	54.6% (395)	0.4	0.1	49.5% (48)	56.5% (329)	0.2	0.14
Diabetes mellitus	29.8% (245)	28.9% (28)	30% (217)	0.9	0.03	28.9% (28)	31.2% (181)	0.7	0.05
Arterial hypertension	81% (665)	82.5% (80)	80.8% (585)	0.8	0.04	82.5% (80)	79.4% (462)	0.6	0.08
Prior Stroke	9.7% (80)	13.4% (13)	9.3% (67)	0.3	0.13	13.4% (13)	8.8% (51)	0.2	0.15
Pacemaker	29.8% (245)	30% (29)	36.3% (216)	0.5	0.001	30% (29)	32.5% (189)	0.6	0.06
*CRT*	*14.4% (118)*	*19.6% (19)*	*13.7% (99)*	*0.2*	*0* *.* *16*	*19.6% (19)*	*16.5% (96)*	*0.5*	*0.08*
*+ ICD*	*22.3% (183)*	*32% (31)*	*21% (152)*	***0.02***	*0* *.* *25*	*32% (31)*	*26.1% (152)*	*0.3*	*0.13*
Atrial fibrillation	74.1% (608)	68% (66)	74.9% (542)	0.4	0.11	68% (66)	74.4% (433)	0.4	0.1
GFR (mL/Min)	50 ± 25	49 ± 26	50 ± 25	0.7	0.05	49 ± 26	51 ± 27	0.6	0.05
NT-proBNP (ng/L)*	2,664 (947–5,973)	2,838 (1,111–5,319)	2,649 (933–6,072)	1	0.05	2,838 (1,111–5,319)	2,702 (888–6,531)	0.9	0.02
LVEF (%)	42 ± 15	41 ± 16	42 ± 15	0.8	0.02	41 ± 16	40 ± 15	0.5	0.07
TR grade III	18.6% (153)	22.7% (22)	18.1% (131)	0.3	0.11	22.7% (22)	17.9% (104)	0.3	0.12
LVEDD (mm)	57 ± 9	59 ± 9	56 ± 9	0.06	0.25	59 ± 9	57 ± 9	0.2	0.18
LA diameter (mm)	46 ± 9	46 ± 9	46 ± 9	0.9	0.01	46 ± 9	46 ± 9	0.8	0.03
Degenerative MR etiology	35.7% (293)	26.8% (26)	36.9% (267)	0.1	0.22	26.8% (26)	27.3% (159)	1	0.01
Functional MR etiology	52.6% (432)	58.8% (57)	51.8% (375)			58.8% (57)	58.6% (341)		
Mixed MR etiology	11.7% (96)	14.4% (14)	11.3% (82)			14.4% (14)	14.1% (82)		
Median procedure duration (min)*	80 (55–115)	112 (80–142)	77 ([54–109)	**<0** **.** **001**	0.57	112 (80–142)	80 (55–112)	**<0** **.** **001**	0.53
mTEER device									
* MitraClip©*	*79.2% (650)*	*81.4% (79)*	*78.9% (571)*	*0*.*6*	*0*.*07*	*81.4% (79)*	*81.1% (472)*	*1*	*0*.*009*
* PASCAL™*	*20.8% (171)*	*18.6% (18)*	*21.1% (153)*			*18.6% (18)*	*18.9% (110)*		
Number of clips implanted*	1 (1–2)	2 (1–2)	1 (1–2)	**<0** **.** **001**	0.63	2 (1–2)	1 (1–2)	**<0** **.** **001**	0.63
Periprocedual MR reduction (carpentier grade)	*Δ*2.0 ± 0.6	*Δ*1.0 ± 0.4	*Δ*2.2 ± 0.4	**<0** **.** **001**	2.9	*Δ*1.0 ± 0.4	*Δ*2.2 ± 0.4	**<0** **.** **001**	2.9
Length of hospital stay (days)*	6 (4–9)	7 (5–11(	6 (4–9)	**0.003**	0.15	7 (5–11)	6 (4–9)	**0** **.** **009**	0.11
Heart Failure Medication									
ACE-/AT1 Inhibitors	72.2% (593)	71.1% (69)	72.9% (524)	0.8	0.04	71.1% (69)	73% (421)	0.7	0.04
ARN Inhibitor	13.5% (111)	14.4% (14)	13.5% (97)	0.9	0.03	14.4% (14)	13.4% (77)	0.7	0.03
Beta Blockers	88.8% (729)	87.6% (85)	89.4% (644)	0.7	0.06	87.6% (85)	89.6% (518)	0.6	0.06
Diuretics	92.8% (762)	89.7% (87)	93.2% (675)	0.8	0.13	89.7% (87)	93.6% (545)	0.2	0.14
Aldosteron antagonists	48.2% (396)	46.4% (45)	48.8% (351)	0.7	0.05	46.4% (45)	51% (294)	0.4	0.09
SGLT-II-inhibitors	4.8% (39)	4.1% (4)	4.8% (35)	1	0.03	4.1% (4)	5% (29)	1	0.04
Vericiguat	0.1% (1)	0% (0)	0.1% (1)	1	0.05	0% (0)	0.2% (1)	1	0.06

Data presented as absolute and relative frequencies or means ± standard deviations (SDs).

ACE, angiotensin-convertingenzyme. ARN, angiotensin-receptor-neprilysin. AT1, angiotensin-1-receptor. BMI, body mass index. CAB-OP, coronary artery bypass-operation. CAD, coronary artery disease. COPD, chronic obstructive pulmonary disease. CRT, cardiac resynchronization therapy. GFR, glomerular filtration rate. ICD, implantable cardioverter defibrillator. LA, left atrium. LVEDD, left ventricular enddiastolic diameter. LVEF, left ventricular ejection fraction. MR, mitral valve regurgitation. mTEER, transcatheter edge-to-edge mitral valve repair. NYHA, New-York-Heart-Association. PCI, percutaneous coronary intervention. SGLT, sodium-glucose linked transporter. TR, tricuspid valve regurgitation.

Bold values indicate the statistical significance of the provided *p*-value.

* Data presented as the median and interquartile range (25th – 75th percentile).

# *p*-value comparing the observed frequencies between patients with and without residual MR ≤ I° after mTEER.

◊ standardized mean difference comparing the balance of covariates between patients with and without residual MR ≤ I° after mTEER before and after propensity-score-matching.

**Table 2:** Short-term outcomes including MACCEs of patients with or without residual MR ≤ I° after mTEER.

Variable	Overall cohort	Residual MR II° after mTEER	Residual MR I° after mTEER	*p*-value^#^
	(*n*=821)	(*n*=97)	(*n*=724)	
Stroke	0.6% (5)	0% (0)	0.7% (5)	1
Myocardial infarction	0% (0)	0% (0)	0% (0)	1
Bleeding complications	3.2% (26)	6.2% (6)	2.8% (20)	0.1
*MVARC I*	*2.1%* (*17*)	*5.2%* (*5*)	*1.7%* (*12*)	
*MVARC II*	*0.6%* (*5*)	*1.0%* (*1*)	*0.6%* (*4*)	
*MVARC III*	*0.1%* (*1*)	*0%* (*0*)	*0.1%* (*1*)	*0.2*
*MVARC IV*	*0.4%* (*3*)	*0%* (*0*)	*0.4%* (*3*)	
*MVARC V*	*0%* (*0*)	*0%* (*0*)	*0%* (*0*)	
Cardiac conduction system disturbances	0% (0)	0% (0)	0% (0)	1
In-hospital mortality	3.5% (29)	5.2% (5)	3.3% (24)	0.4
*Cardiac cause*	*2.2%* (*18*)	*2.1%* (*2*)	*2.2%* (*16*)	*1*
*Non-cardiac cause*	*1.3%* (*11*)	*3.1%* (*3*)	*1.1%* (*8*)	*0.8*

Data presented as absolute and relative frequencies.

MR, mitral valve regurgitation; mTEER, transcatheter edge-to-edge mitral valve repair; MVARC, mitral valve academy research consortium; MACCEs, major adverse cardiovascular and cerebrovascular events.

Italic values indicate subgroups of the above cited variable.

# *p*-value comparing the observed frequencies between patients with and without residual MR ≤ I° after mTEER.

### Predictors of mild residual MR after mTEER

In the univariable logistic regression analysis, previous ICD implantation [odds ratio (OR) 0.56, 95%–confidence interval (95%–CI) 0.36–0.91, *p* = 0.02], age <65 years at the time of the procedure (OR 0.39, 95%–CI 0.2–0.78, *p* = 0.005), and a left ventricular enddiastolic diameter (LVEDD) > 62 mm prior to mTEER (OR 0.52, 95–CI 0.31–0.88, *p* = 0.01) were identified as factors which were associated with residual MR of II°. After adjustment was conducted in the subsequent multivariable logistic regression model, age <65 years at the time of procedure (OR 0.39, 95%–CI 0.19–0.84, *p* = 0.01) persisted as a significant inverse predictor of residual MR ≤ I°. The results of the univariable and multivariable logistic regression analyses are presented in Tables 3, 4, respectively.

**Table 3:** Independent predictors of residual MR ≤ I° after mTEER in the univariable logistic regression analysis.

Variable	Odds ratio	95%-Confidence interval	*p*-value^#^
Age	0.97	0.95–0.998	**0** **.** **03**
***Age < 65 years***	*0* *.* *39*	*0.2–0.78*	***0*** ***.*** ***005***
ICD	0.56	0.36–0.91	**0** **.** **02**
LVEDD before mTEER	0.97	0.94–0.99	**0** **.** **04**
***LVEDD > 62 mm before mTEER***	*0* *.* *52*	*0.31–0.88*	***0*** ***.*** ***01***

Data presented as ratios with the corresponding confidence intervals.

ICD, implantable cardioverter defibrillator; LVEDD, left ventricular enddiastolic diameter; MR, mitral valve regurgitation; mTEER, transcatheter edge-to-edge mitral valve repair.

Bold values indicate the statistical significance of the provided *p*-value.

# *p*-value indicating the significance of the association between the presented value and a residual MR ≤ I° after mTEER.

**Table 4:** Independent predictors of residual MR ≤ I° after mTEER in the multivariable logistic regression analysis.

Variable	Odds ratio	95%-Confidence interval	*p*-value^#^
Age	0.98	0.94–0.99	**0** **.** **03**
***Age < 65 years***	*0* *.* *39*	*0.19–0.84*	***0*** ***.*** ***01***

Data presented as ratio with the corresponding confidence interval.

MR, mitral valve regurgitation; mTEER, transcatheter edge-to-edge mitral valve repair.

Bold values indicate the statistical significance of the provided *p*-value.

# *p*-value indicating the significance of the association between the presented value and a residual MR ≤ I° after mTEER.

### Propensity-score-matched analysis of long-term survival

In PSM analysis, improvements were made in the balance of clinical characteristics, which significantly differered between the two cohorts before matching. The aforementioned differences in procedural characteristics between cohorts remained significant, as also demonstrated in Table 1. With respect to long-term survival, patients with residual MR ≤ I° did not ignificantly differ from those with residual MR of II° three years after mTEER in the overall cohort [54.6% (395/724) vs. 53.9% (52/97), *p* = 0.4]. This observation in the survival rate three years after mTEER between both cohorts was subsequently corroborated after adjustment using PSM [54.9% (320/582) vs. 53.9% (52/97), *p* = 0.4]. Univariable Cox regression analysis revealed that neither residual MR ≤ I° [hazard ratio (HR) 0.86, 95%–CI 0.63–1.18, *p* = 0.4] nor residual MR II° (HR 1.16, 95%–CI 0.85–1.59, *p* = 0.4) was a predictor of mortality in the overall cohort. According to the multivariable Cox regression analysis results, the following factors were identified as independent predictors of mortality: male sex (HR 1.3, 95%–CI 1.05–1.7, *p* = 0.02), chronic obstructive pulmonary disease (COPD) (HR 1.5, 95%–CI 1.1–1.9, *p* = 0.003), NYHA class IV (HR 1.4, 95%–CI 1.1–1.9, *p* = 0.005), concomitant severe tricuspid regurgitation (TR) (HR 1.8, 95%–CI 1.4–2.4, *p* < 0.001), and chronic kidney disease with a glomerular filtration rate (GFR) < 30 mL/min (HR 1.4, 95%–CI 1.1–1.8, *p* = 0.01).

After additional stratification of the patients according to MR etiology following PSM, a diminished survival three years after mTEER was observed among patients with mixed MR etiology and residual MR of II° compared to patients with residual MR ≤ I° [38.6% (5/14) vs. 49.9% (41/82), *p* = 0.04]. No significant differences were observed in survival three years after mTEER between both cohorts in the subsets of degenerative [57.6% (15/26) vs. 71.6% (114/159), *p* = 0.2] and functional MR etiology [55.2% (31/57) vs. 50% (171/341), *p* = 0.6], respectively.

**Figure 2:**
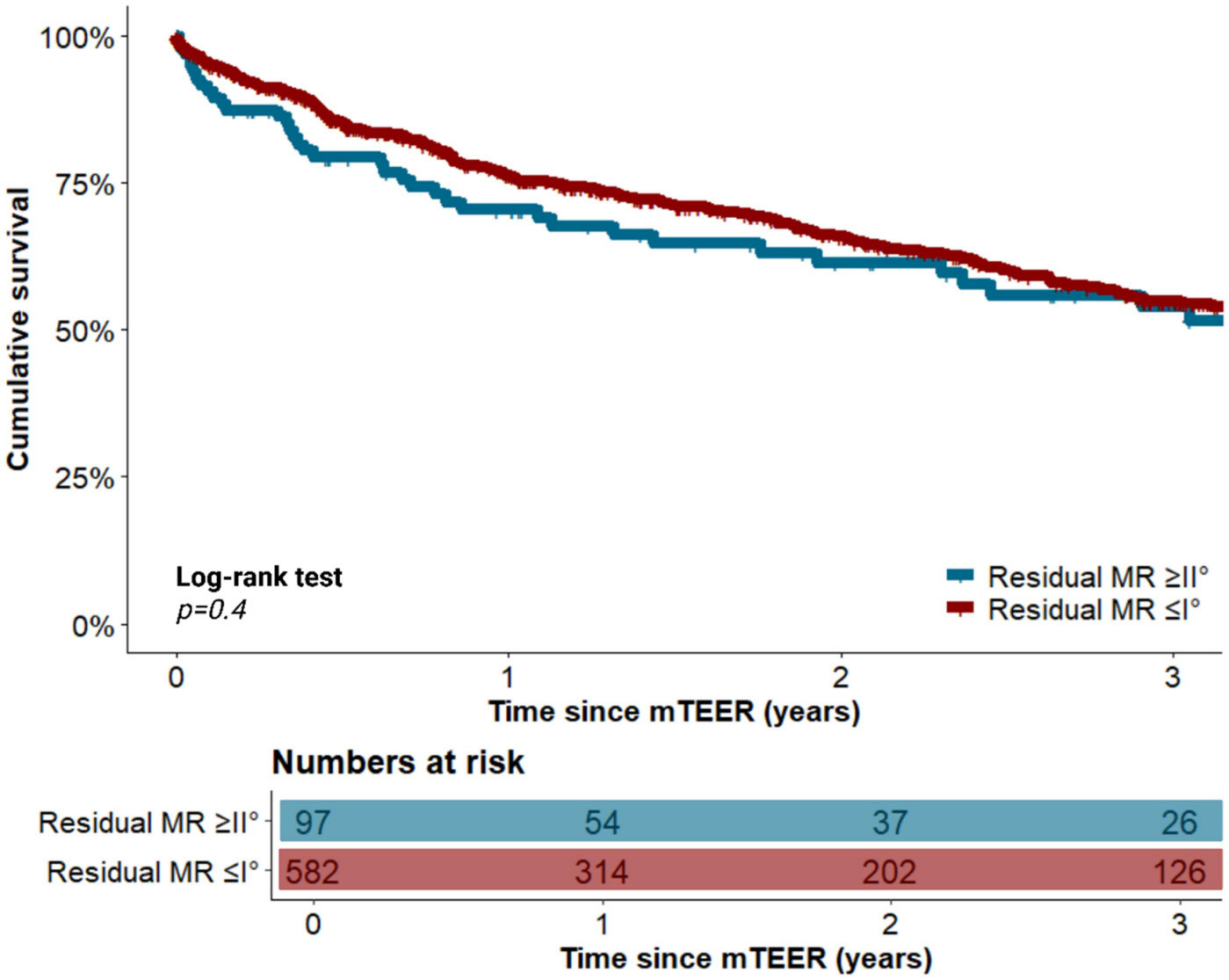
Long-term survival of patients with and without residual MR ≤ I° following mTEER after propensity-score-matching. MR, mitral valve regurgitation; mTEER, transcatheter edge-to-edge mitral valve repair.

The significant mortality predictors that were identified in the univariable and multivariable Cox regression analyses are listed in the Supplementary Tables S3, S4, respectively**.** The course of long-term survival after mTEER is shown before PSM in Supplementary Figure S1, after PSM in Figure 2, and after additional stratification according to MR etiology in Supplementary Figure S2.

## Discussion

In the current era, characterized by the increasing significance of transcatheter valve interventions, the selection and referral of suitable candidates remain topics of considerable debate, with the objective of ensuring optimal clinical outcomes. In the present multicenter study cohort revealed, MR of II° was present in 11.8% (97/821) of the patients. Patients with residual MR of II° exhibited higher morbidity and longer procedure-related parameters, suggesting increased MR complexity. However, mTEER was equally effective and a difference in long-term survival compared to patients with residual MR ≤ I° was only observed in the subset of patients with mixed MR etiology.

Notably, except for residual MR of II°, no moderate-to-severe residual MR could be identified in the present cohort. These findings differ from those of previous studies, which reported a prevalence of moderate-to-severe residual MR in 4.7%–30% of mTEER patients across various MR etiologies (10–14). One potential explanation for these findings is the premature termination of the procedure in 5.4% (47/868) of the patients in the present study because of inadequate leaflet grasping. In light of the projected insufficient reduction success in MR success, these patients could have been designated as patients with residual moderate-to-severe MR if clip application had proceeded.

In addition to the aforementioned limitations, the comparability of the results between this study and previous studies remains challenging. This limitation is due to the lack of comparable echocardiographic characteristics of MR morphology (10–14). Therefore, additional factors, such as calcification of the mitral valve annulus and leaflets (26), coaptation area (27) and multiple and eccentric regurgitant jets (28), may have contributed significantly to the inadequate reduction of MR in previous studies compared with this study.

A significantly more challenging MR morphology can also be assumed in patients with residual MR of II° in this study because of the longer procedure times and the higher clip implantation rates. Despite the intensified efforts, a reduction in residual MR ≤ I° remained unsuccessful. The clinical challenge was further compounded by the higher morbidity of patients with residual MR of II°, as evidenced by an increased prevalence of NYHA class IV and a greater prevalence of implanted ICDs. Consequently, despite being significantly younger than the remaining cohort, these patients were still deemed unsuitable candidates for surgical mitral valve repair. However, the mTEER procedure was equally feasible, as short-term complication and MACCE rates were comparable between both cohorts.

The clinical cohort characteristics of this study were generally comparable to those of current multicenter registries, including the ACCESS-EU registry (29), the Getting Reduction of mitrAl inSufficiency by Percutaneous clip implantation in ITaly (GRASP-IT) registry (30), the European Registry of Transcatheter Repair for Secondary Mitral Regurgitation (EuroSMR) (31) and the German transcatheter mitral valve interventions (TRAMI) registry (32). With respect to the major landmark studies on mTEER, patients in this study were older than those in the EVEREST II (7) and COAPT studies (6) were. A comparison of the present study with the Randomized Investigation of the MitraClip Device in Heart Failure: 2nd Trial in Patients with Clinically Significant Functional Mitral Regurgitation (RESHAPE-HF2) study (8) revealed no significant disparities in clinical characteristics between the cohorts, as previously reported (33). Due to limited data availability in the present cohort, no further echocardiographic parameters of left heart volumes, right heart function, and pulmonary artery pressures could be analyzed, rendering a comparison of these parameters with those of the major landmark studies impossible.

This study contributes to the existing body of evidence through the identification of inverse preprocedural predictors of a successful reduction in MR to ≤I° using mTEER. With respect to age at the time of the procedure, a study by El-Shurafa et al. also demonstrated a younger age among patients with residual MR, although this trend did not reach statistical significance (10). The relatively young age of the patients, defined as those <65 years of age, constitutes a contentious issue. Current guidelines recommend surgical mitral valve repair for this group (4). However, given that these patients were, by definition, deemed unsuitable candidates for surgery due to the heightened perioperative risk, the increased morbidity of this cohort could have caused a therapeutic challenge, resulting in a subsequent increase in the incidence of residual MR II°. The LVEDD > 62 mm could be interpreted as a marker of advanced ventricular remodeling, which may have hindered the reduction success because of the consecutive increase in annulus dilatation. Within this context, Buzzatti et al. reported significantly higher LVEDD values in patients with functional MR (who are largely affected by ventricular remodeling) with residual moderate-to-severe MR than in patients with residual mild MR (11). Preprocedural ICD implantation does not seem, in our opinion, to directly interfere with the success of MR reduction, but seems to constitute a further surrogate parameter of advanced congestive heart failure, indicating a patient population whose increased risk of sudden cardiac death requires device therapy even prior to mTEER.

With respect to long-term survival, no significant association was observed for the severity of residual MR in the overall cohort even after adjustment of covariates through PSM. Further stratification of matched patients according to MR etiology revealed that in patients with mixed MR etiology, survival was worse in those with residual MR II° compared to residual MR ≤ I°. However, given the considerably small sample size in this subgroup, this result should be considered with extreme caution and confirmed by further prospective studies. The previous growing body of research has demonstrated that the severity of residual MR has a deleterious effect on survival, as evidenced by studies of both the overall cohort (11) and in specific subcohorts, including patients with degenerative MR (11–13), functional MR (11), and atrial-functional MR (14). However, given that the study cohorts also included patients with residual moderate-to-severe MR and residual severe MR after mTEER, the identified benign effect of residual MR ≤ I° on mortality may be attributed to the comparison with these patients. Preliminary findings indicate that a residual MR of II° in isolation appears to have no significant effect on long-term survival. However, whether patients with residual MR ≤ I° in this study could benefit more than patients with residual MR of II° in terms of improvement in congestive heart failure symptoms and hospitalization rates remains unclear. This is due to the proven effectiveness of mTEER in these areas, which affects the clinical outcome (8, 9). Furthermore, the extent to which patients with residual MR II° in this study are particularly susceptible to further progression of MR remains to be elucidated. This, in turn, could impair heart failure–related symptoms, hospitalization rates, and even survival. Buzzatti et al. identified residual moderate-to-severe MR as a predictor of subsequent progression in MR severity during follow-up (11). Regrettably, this study was unable to conduct additional analyses of long-term outcomes because the amount of relevant data was insufficient.

The findings, derived from this study, suggest that the higher morbidity observed in patients with a lower mean age is the primary factor contributing to the less successful MR reduction. While this did not appear to negatively influence long-term survival rates in the overall cohort, the quality of the treatment outcome could be reduced by the persistent burden of symptoms and the renewed progression of MR, necessitating rehospitalization and reintervention. In light of the findings from the multivariable logistic regression analysis, which indicated that age <65 years emerged as the predominant inverse predictor of residual MR of ≤I°, it is vital to critically assess suitable candidates, particularly within this relatively younger cohort of patients, given their potentially higher life expectancy attributed to their youth.

## Limitations

Several limitations should be considered with regard to the interpretation of the results. The retrospective design of the study precludes conclusions regarding potential causalities. Additionally, the potentially limited statistical power due to the comparatively small sample size of patients with residual MR of II° should be taken into account. The possibility of interobserver bias in the graduation of the MR cannot be entirely dismissed, as the echocardiography laboratories at the respective centers were responsible for the analysis and no core echocardiography laboratory could be involved retrospectively. Although the absence of significant mitral valve stenosis with a transmitral gradient >5 mmHg after mTEER was defined as part of procedural success, no further detailed recordings of the transmitral gradients were conducted before and after mTEER. Consequently, a direct comparison between this study and mTEER studies that examined the transmitral gradient and left atrial pressures as an additional determinants of the outcome was not feasible (12, 13, 34, 35). The limited availability of data also prevented the analysis of other parameters of LV volume apart from LVEDD, right heart function, and pulmonary artery pressures. This limits the comparability with the major landmark studies such as COAPT, Mitra-FR, and RESHAPE-HF2. Moreover, it was not possible to incorporate these factors into the PSM model or the regression analyses, leaving a risk of residual bias in the results. Given the limitations of the available data, it was further only possible to investigate the influence of residual MR on long-term outcomes after mTEER. The extent to which the long-term progression of residual MR to recurrent MR would have influenced the results remains unclear. The same applies to the impact of heart-failure related hospitalizations after mTEER on long-term outcomes.

## Conclusion

Residual MR of II° was observed in 11.8% (97/821) of patients who underwent mTEER. They were significantly younger and more morbid than patients with residual MR ≤ I°. Although no significant decrease in survival was observed in patients with residual MR of II° in the overall cohort before and after adjustment via PSM, future studies should elucidate potential differences in the symptom burden of congestive heart failure, hospitalization rates and progression rates of MR. The identified preprocedural inverse predictors of residual MR ≤ I° can provide guidance in the screening and referral of current “real-world” mTEER candidates with careful consideration of the study design and the associated limitations.

## Data Availability

The raw data supporting the conclusions of this article will be made available by the authors, without undue reservation.
