# Supplementary material for: Impact of residual moderate mitral regurgitation after transcatheter edge-to-edge repair on long-term survival: insights from a multicenter cohort study
Source: Front Cardiovasc Med. 2026 Feb 18;13:1720322. doi: 10.3389/fcvm.2026.1720322 (PMC12958024; doi:10.3389/fcvm.2026.1720322)
Supplement: Supplementary file 1 [file Datasheet1.docx]

**Supplementary Materials**

**Supplementary Table S1: Echocardiographic criteria of MR graduation before and after mTEER**

| Before mTEER | After mTEER | | |
| --- | --- | --- | --- |
| Severe MR (III°) | **Severe residual MR (III°)** | **Moderate residual MR (II°)** | **Mild/minimal residual MR (≤I°)** |
| Qualitative | **Qualitative** | | |
| Large coaptation defect | Large coaptation defect |  |  |
| Large central jet | Large central jet |  |  |
| Large holosystolic convergence zone | Large holosystolic convergence zone |  |  |
| Semi-quantitative | **Semi-quantitative** | | |
| V. contracta ≥7mm | V. contracta ≥7mm | V. contracta 3-6mm | V. contracta <3mm |
| Pulmonary vein systolic flow reversal | Pulmonary vein systolic flow reversal |  |  |
| E-wave >1,2 m/s | E-wave >1,2 m/s |  |  |
| Quantitative | **Quantitative** | | |
| EROA ≥40 cm²^,*^ | EROA ≥40 cm²^,*^ | EROA 20-39 cm²^,#^ | EROA <20cm² |
| Regurgitant volume ≥60mL^*^ | Regurgitant volume ≥60mL^*^ | Regurgitant volume 30-59mL^#^ | Regurgitant volume <30mL |
| Regurgitant fraction ≥50% | Regurgitant fraction ≥50% | Regurgitant fraction 30-49% | Regurgitant fraction <30% |

^*^EROA ≥30 cm² and regurgitant volume ≥45 mL as markers of severe MR in case of an elliptical regurgitant orifice

^#^EROA 20-29 cm² and regurgitant volume 30-44mL as markers of moderate MR in case of an elliptical regurgitant orifice

EROA – effective regurgitant orifice area. MR – mitral valve regurgitation. mTEER – transcatheter edge-to-edge mitral valve repair. V. contracta – Vena contracta.

**Supplementary Table S2: Clinical and procedural characteristics of patients with and without a loss of follow-up**

| Variable | Patients not lost to FU  (n=766) | Patients lost to FU  (n=55) | p-value^#^ |
| --- | --- | --- | --- |
| Age (years) | 78 ± 8 | 79 ± 7 | 0.1 |
| euroSCORE II (%)* | 16.1%  [9 – 29.8] | 12.4%  [5.5 – 16.2] | 0.3 |
| STS Risk Score (%)* | 6.7%  [4 – 12.4] | 5.8%  [3.8 – 9] | 0.1 |
| Male sex | 61.9% (474) | 72.7% (40) | 0.1 |
| NYHA class I  NYHA class II  NYHA class III  NYHA class IV | 0.1% (1)  3.4% (26)  75.8% (581)  20.6% (158) | 0% (0)  0% (0)  72.7% (40)  27.3% (15) | 0.3 |
| COPD | 18.3% (140) | 10.9% (6) | 0.2 |
| CAD | 62.4% (478) | 60% (33) | 0.8 |
| Prior CAB-OP | 28.5% (218) | 18.2% (10) | 0.1 |
| Prior PCI | 53.9% (413) | 54.5% (30) | 1 |
| Pacemaker  *CRT*  *+ ICD* | 29.6% (227)  *14.4% (110)*  *22.5% (172)* | 27.3% (15)  *14.5% (8)*  *20% (11)* | 0.8  *1*  *0.7* |
| Diabetes mellitus | 30.4% (233) | 21.8% (12) | 0.2 |
| Arterial hypertension | 81.5% (624) | 74.5% (41) | 0.2 |
| Prior Stroke | 9.4% (72) | 14.5% (8) | 0.2 |
| LVEF (%) | 41 ± 15 | 44 ± 14 | 0.3 |
| Atrial fibrillation | 73.9% (566) | 76.4% (42) | 0.8 |
| GFR (mL/Min) | 50 ± 26 | 53 ± 19 | 0.3 |
| NT-proBNP (ng/L)* | 2299  [605 – 5528] | 2050  [431 – 5665] | 0.6 |
| TR grade III | 17.9% (137) | 29.1% (16) | 0.06 |
| Degenerative MR etiology  Functional MR etiology  Mixed MR etiology | 36.9% (278)  52.5% (402)  11.2% (86) | 27.3% (15)  54.5% (30)  18.2% (10) | 0.1 |
| Median procedure duration (min)* | 82  [55 – 117] | 68  [50 – 84] | 0.1 |
| Number of clips implanted* | 1 [1 – 2] | 1 [1 – 1] | 0.3 |
| Periprocedual MR reduction  (Carpentier grade) | Δ2.0 ± 0.6 | Δ2.0 ± 0.3 | 0.6 |
| Length of hospital stay (days)* | 7 [4 – 9] | 5 [3 – 7] | 0.1 |
| Overall-MACCE  *Cerebral/systemic thromboemb. event*  *Bleeding requiring intervention*  *In-hospital death from cardiovasc. cause* | 5.6% (43)  *0.7% (5)*  *3.3% (25)*  *2.2% (17)* | 3.6% (2)  *0% (0)*  *1.8% (1)*  *1.8% (1)* | 0.8  *1*  *1*  *1* |
| In-hospital death from any cause | 3.7% (28) | 1.8% (1) | 0.7 |
| Heart Failure Therapy |  |  |  |
| ACE-/AT1 Inhibitors | 72.7% (557) | 65.5% (36) | 0.3 |
| ARN Inhibitor | 13.1% (100) | 20% (11) | 0.2 |
| Beta Blockers | 89.2% (683) | 83.6% (46) | 0.3 |
| Diuretics | 93.1% (713) | 89.1% (49) | 0.5 |
| Aldosterone antagonists | 48.4% (371) | 45.5% (25) | 0.7 |
| SGLT-II-Inhibitors | 4.6% (35) | 7.3% (4) | 0.5 |
| Vericiguat | 0% (0) | 1.8% (1) | 0.07 |

Data presented as absolute and relative frequencies or means ± standard deviations (SDs). * - Data presented as the median and interquartile range [25^th^ – 75^th^ percentile].^#^ - p-value comparing the observed frequencies between patients with and without a loss of follow-up. ACE – angiotensin-converting-enzyme. ARN – angiotensin-receptor-neprilysin. AT1 – angiotensin-1-receptor. CAB-OP – coronary artery bypass-operation. CAD – coronary artery disease. COPD – chronic obstructive pulmonary disease. CRT – cardiac resynchronization therapy. FU – follow-up. GFR – glomerular filtration rate. ICD – implantable cardioverter defibrillator. LVEF – left ventricular ejection fraction. MACCE – major adverse cardiovascular and cerebrovascular events. MR – mitral valve regurgitation. mTEER – transcatheter edge-to-edge mitral valve repair. NYHA – New-York-Heart-Association. PCI – percutaneous coronary intervention. SGLT – sodium-glucose linked transporter. TR – tricuspid valve regurgitation.

**
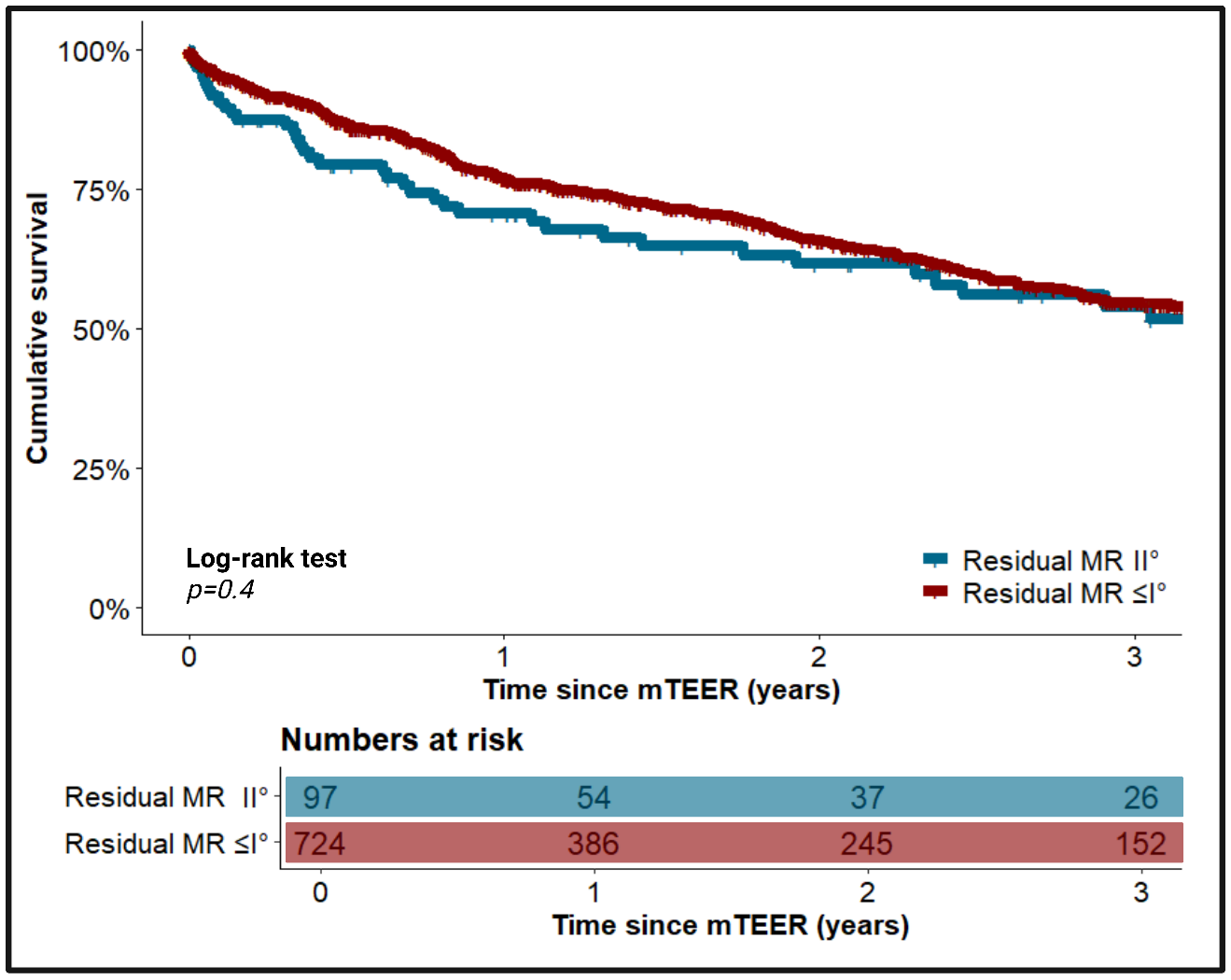
**

**Supplementary Figure S1: Long-term survival of patients with and without residual MR ≤I° after mTEER**

MR – mitral valve regurgitation. mTEER – transcatheter edge-to-edge mitral valve repair.

**Supplementary Table S3: Independent predictors of mortality after univariable Cox regression analysis**

| Variable | Hazard ratio | 95%-Confidence interval | p-value^#^ |
| --- | --- | --- | --- |
| Male sex | 1.4 | 1.08-1.75 | **0.009** |
| COPD | 1.64 | 1.26-2.1 | **<0.001** |
| CAD | 1.28 | 1.03-1.63 | **0.04** |
| CRT | 1.29 | 0.96-1.74 | 0.09 |
| ICD | 1.3 | 1.05-1.7 | **0.02** |
| NYHA class IV | 1.67 | 1.3-2.16 | **<0.001** |
| TR grade III | 1.9 | 1.47-2.47 | **<0.001** |
| Atrial fibrillation | 1.3 | 1.01-1.7 | **0.05** |
| GFR < 30 mL/min | 1.53 | 1.17-2 | **0.001** |

Data presented as ratios with the corresponding confidence intervals. ^#^ - p-value indicating the

significance of the association between the presented value and mortality after mTEER. CAD – coronary artery disease. COPD – chronic obstructive pulmonary disease. CRT – cardiac resynchronization therapy. GFR – glomerular filtration rate. ICD – implantable cardioverter defibrillator. mTEER – transcatheter edge-to-edge mitral valve repair. NYHA – New-York-Heart-Association. TR – tricuspid valve regurgitation.

**Supplementary Table S4: Independent predictors of mortality after multivariable Cox regression analysis**

| Variable | Hazard ratio | 95%-Confidence interval | p-value^#^ |
| --- | --- | --- | --- |
| Male sex | 1.3 | 1.05-1.7 | **0.02** |
| COPD | 1.5 | 1.1-1.9 | **0.003** |
| NYHA class IV | 1.4 | 1.1-1.9 | **0.005** |
| TR grade III | 1.8 | 1.4-2.4 | **<0.001** |
| GFR < 30 mL/min | 1.4 | 1.1-1.8 | **0.01** |

Data presented as ratios with the corresponding confidence intervals. ^#^ - p-value indicating the

significance of the association between the presented value and mortality after mTEER. COPD – chronic obstructive pulmonary disease. GFR – glomerular filtration rate. mTEER – transcatheter edge-to-edge mitral valve repair. NYHA – New-York-Heart-Association. TR – tricuspid valve regurgitation.


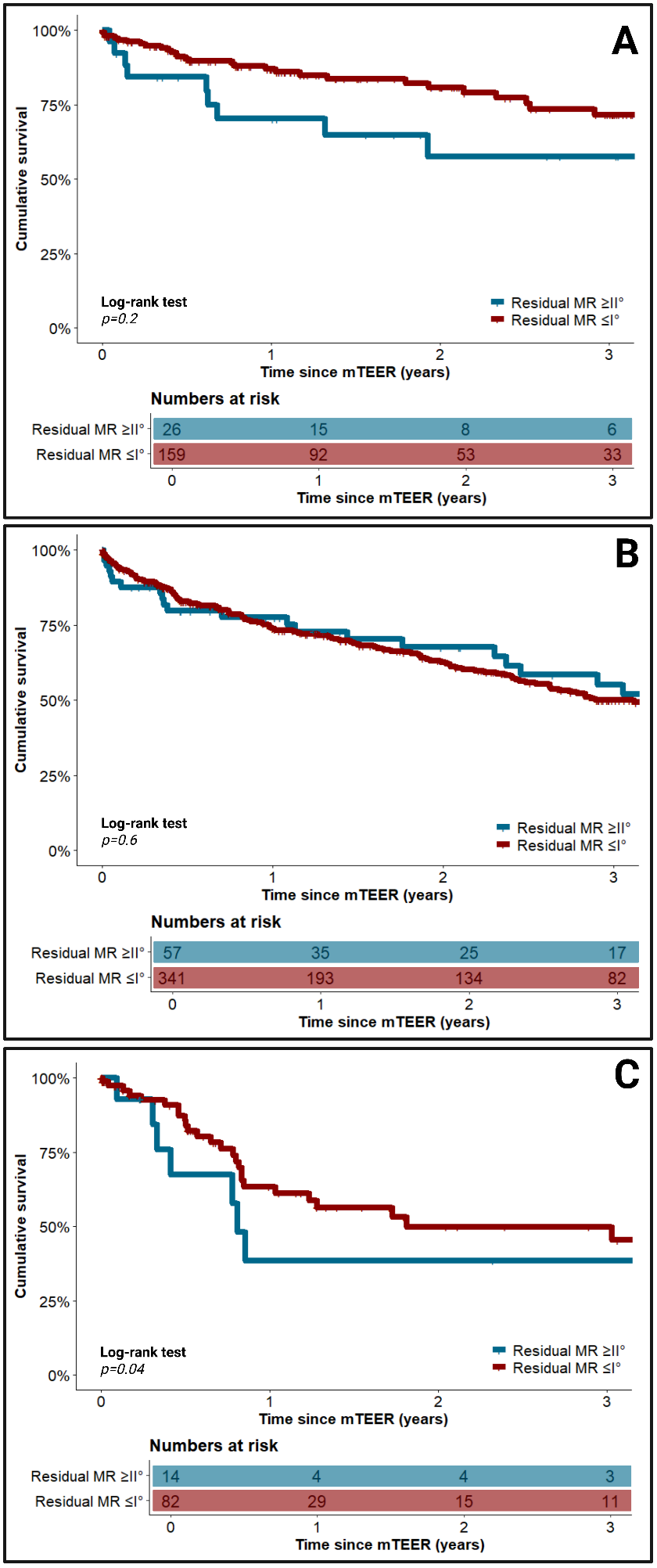


**Supplementary Figure S2:** **Long-term survival of patients with and without residual MR ≤I° following mTEER after propensity-score-matching and additional stratification according to MR etiology (A: Degenerative MR etiology, B: Functional MR etiology, C: Mixed MR etiology)**

MR – mitral valve regurgitation. mTEER – transcatheter edge-to-edge mitral valve repair.
